# Targeting the IDO1 pathway in cancer: from bench to bedside

**DOI:** 10.1186/s13045-018-0644-y

**Published:** 2018-08-02

**Authors:** Ming Liu, Xu Wang, Lei Wang, Xiaodong Ma, Zhaojian Gong, Shanshan Zhang, Yong Li

**Affiliations:** 1 State Key Laboratory of Respiratory Diseases, Guangzhou Institute of Respiratory Diseases, The First Affiliated Hospital of Guangzhou Medical University, Guangzhou Medical University, Guangzhou, China; 2 Department of Cancer Biology, Lerner Research Institute, Cleveland Clinic, Cleveland, OH USA; 3 Institute for Brain Research and Rehabilitation, South China Normal University, Guangzhou, China; 4 Department of Stomatology, The Second Xiangya Hospital, Central South University, Changsha, China; 5 Department of Stomatology, Xiangya Hospital, Central South University, Changsha, China

**Keywords:** Indoleamine 2, 3-dioxygenases, IDO1, Immunosuppression, Immunotherapy, Clinical trial

## Abstract

Indoleamine 2, 3-dioxygenases (IDO1 and IDO2) and tryptophan 2, 3-dioxygenase (TDO) are tryptophan catabolic enzymes that catalyze the conversion of tryptophan into kynurenine. The depletion of tryptophan and the increase in kynurenine exert important immunosuppressive functions by activating T regulatory cells and myeloid-derived suppressor cells, suppressing the functions of effector T and natural killer cells, and promoting neovascularization of solid tumors. Targeting IDO1 represents a therapeutic opportunity in cancer immunotherapy beyond checkpoint blockade or adoptive transfer of chimeric antigen receptor T cells. In this review, we discuss the function of the IDO1 pathway in tumor progression and immune surveillance. We highlight recent preclinical and clinical progress in targeting the IDO1 pathway in cancer therapeutics, including peptide vaccines, expression inhibitors, enzymatic inhibitors, and effector inhibitors.

## Background

**Fig. 1:**
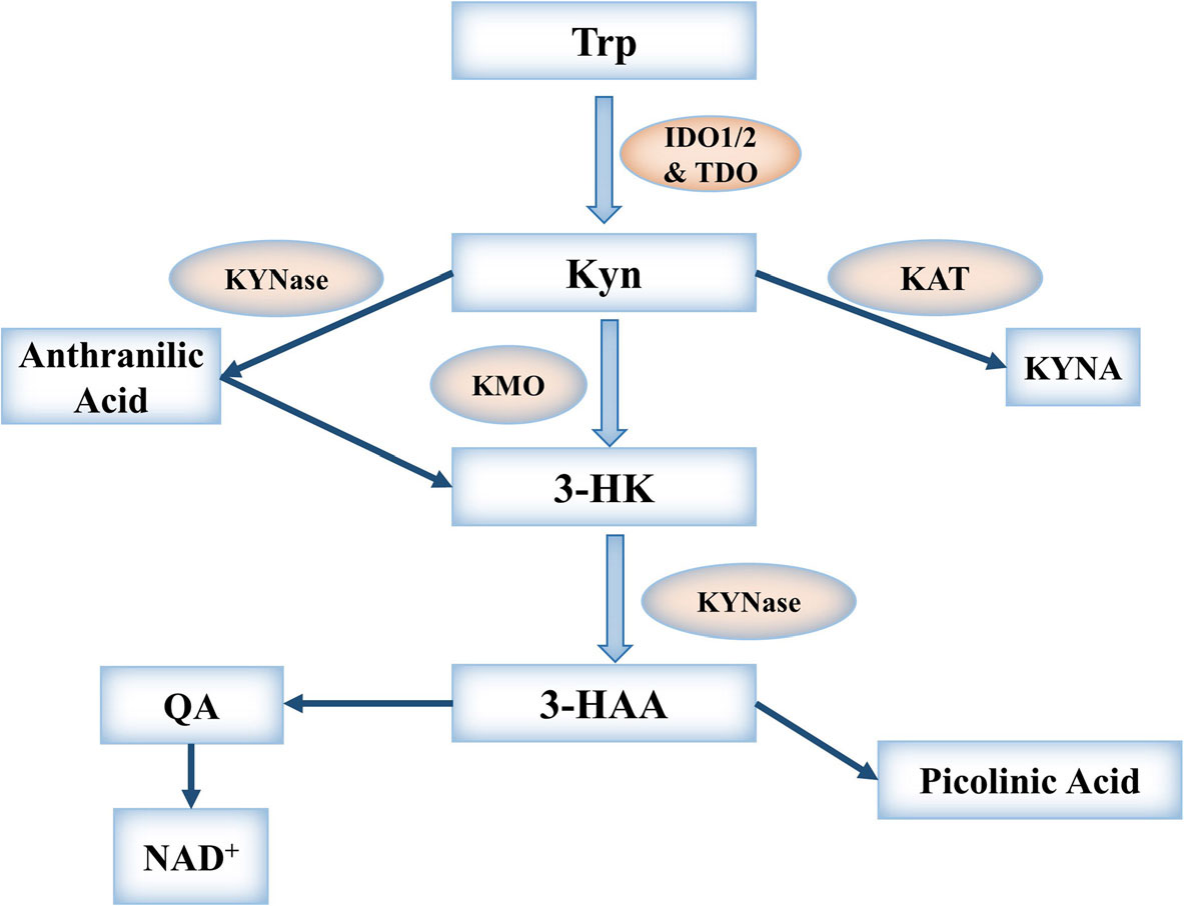
Overview of the IDO metabolic pathway. Approximately 95% of L-tryptophan (Trp) is catabolized into kynurenine (Kyn) through three rate-limiting enzymes: tryptophan 2,3-dioxygenase (TDO) in the liver and indoleamine 2, 3-dioxygenase 1/2 (IDO1/2) in peripheral tissues. Kyn is converted to 3-hydroxykynurenine (3-HK) by kynurenine 3-monooxygenase (KMO), to anthranilic acid (AA) by kynureninase (KYNase), or to kynurenic acid (KYNA) by kynurenine aminotransferase (KAT). Next, catalyzed by KYNase, 3-HK is converted to 3-hydroxyanthranilic acid (3-HAA), which is further converted to quinolinic acid (QA), picolinic acid, nicotinamide adenine dinucleotide (NAD^+^), and other molecules

The tryptophan (Trp) catabolism pathway plays an important role in tumor cell evasion of the innate and adaptive immune systems [1, 2]. Trp is generally utilized in three major metabolic pathways: incorporation into proteins, production of serotonin, and breakdown into kynurenine (Kyn). Kyn is generated via two major routes: in peripheral tissues, controlled by the rate-limiting enzymes indoleamine 2, 3-dioxygenase 1 (IDO1) and indoleamine 2, 3-dioxygenase 2 (IDO2), and the hepatic route, in which tryptophan 2, 3-dioxygenase (TDO) is the rate-limiting enzyme [3] (Fig. 1). Discovered in the 1950s, IDO1 is the most fully characterized enzyme in the Kyn biosynthesis pathway. In healthy post-natal individuals, IDO1 facilitates tolerance by dampening the immune response, whereas during gestation, IDO1 helps protect the fetus from maternal T lymphocytes [4]. On the other hand, IDO1 is a crucial innate immunity regulator that acts by depleting Trp in both the inflammatory and tumor microenvironments [1, 5, 6]. IDO1 is involved in the suppression of effector T and NK cells and differentiation and activation of regulatory T (Treg) cells and myeloid-derived suppressor cells (MDSCs) [7–9]. In addition, IDO1 plays a key role in promoting tumor neovascularization by modulating the expression of interferon-γ (IFN-γ) and interleukin-6 (IL-6) [10, 11]. In prostate cancer cells, IFN-γ and TNF-α-treatment induce IDO1 and IL-6 gene expression [12]. Furthermore, IDO1 is involved in the formation of resistance to immune checkpoint inhibitors [13], and the combination of an IDO1 inhibitor with checkpoint inhibitors represents an alternative strategy in cancer immunotherapy [14].

**Fig. 2:**
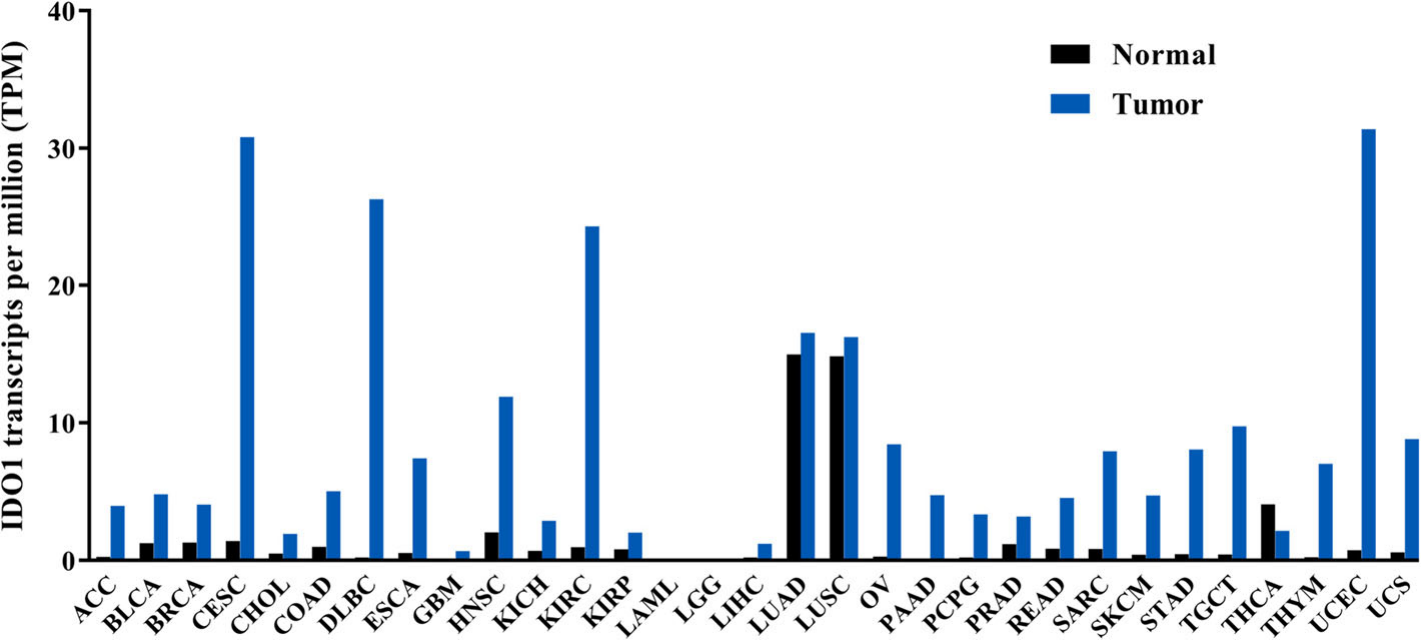
The IDO1 gene transcripts across tumor samples and paired normal tissues. Data are summarized from The Cancer Genome Atlas, https://cancergenome.nih.gov/. The *Y* axis denotes the number of IDO1 transcripts per million of total RNA reads. Abbreviations: ACC, adrenocortical carcinoma; BLCA, bladder urothelial carcinoma; BRCA, breast invasive carcinoma; CESC, cervical squamous cell carcinoma and endocervical adenocarcinoma; CHOL, cholangiocarcinoma; COAD, colon adenocarcinoma; DLBC, lymphoid neoplasm diffuse large B-cell lymphoma; ESCA, esophageal carcinoma; GBM, glioblastoma multiforme; HNSC, head and neck squamous cell carcinoma; KICH, kidney chromophobe; KIRC, kidney renal clear cell carcinoma; KIRP, kidney renal papillary cell carcinoma; LAML, acute myeloid leukemia; LGG, brain lower grade glioma; LIHC, liver hepatocellular carcinoma; LUAD, lung adenocarcinoma; LUSC, lung squamous cell carcinoma; OV, ovarian serous cystadenocarcinoma; PAAD, pancreatic adenocarcinoma; PCPG, pheochromocytoma and paraganglioma; PRAD, prostate adenocarcinoma; READ, rectum adenocarcinoma; SARC, sarcoma; SKCM, skin cutaneous melanoma; STAD, stomach adenocarcinoma; TGCT, testicular germ cell tumors; THCA, thyroid carcinoma; THYM, thymoma; UCS, uterine carcinosarcoma; UCEC, uterine corpus endometrial carcinoma

IDO1 is overexpressed in the vast majority of cancers (Fig. 2, data are summarized from The Cancer Genome Atlas, https://cancergenome.nih.gov/). Several strategies for targeting IDO1 have been assessed in multiple clinical trials and have produced encouraging results. Blockade of IDO1 activity decreased tumor proliferation in a T-lymphocyte-mediated manner and enhanced the efficacy of chemotherapy, radiotherapy, targeted therapy, and immunotherapy [15–19]. In this review, we will first discuss the complex role of IDO1 in regulating the innate and adaptive immune responses. We will then highlight the role of IDO1-related signaling pathways in the tumor microenvironment. Finally, we summarize the current preclinical and clinical studies of IDO-targeting interventions.

## IDO regulatory and effector signaling pathways

Multiple upstream pathways regulate IDO1 expression and function, including the Janus kinase–signal transducer and activator of transcription (JAK–STAT), RAS–protein kinase C (RAS–PKC), nuclear factor kappa-light-chain enhancer of activated B cells (NF-κB), and Kit signaling pathways [20–22]. Downstream of IDO1 are three effector pathways that transduce the effects of IDO1 activity: general control over nonderepressible 2 (GCN2) is activated, mammalian target of rapamycin (mTOR) is inhibited, which is related to Trp deprivation, and the aryl hydrocarbon receptor (AhR) pathway is activated with Kyn as an endogenous AhR ligand [23–25]. These regulatory and effector pathways mediate immunosuppression and neovascularization in the tumor microenvironment.

### Upstream regulators

**Fig. 3:**
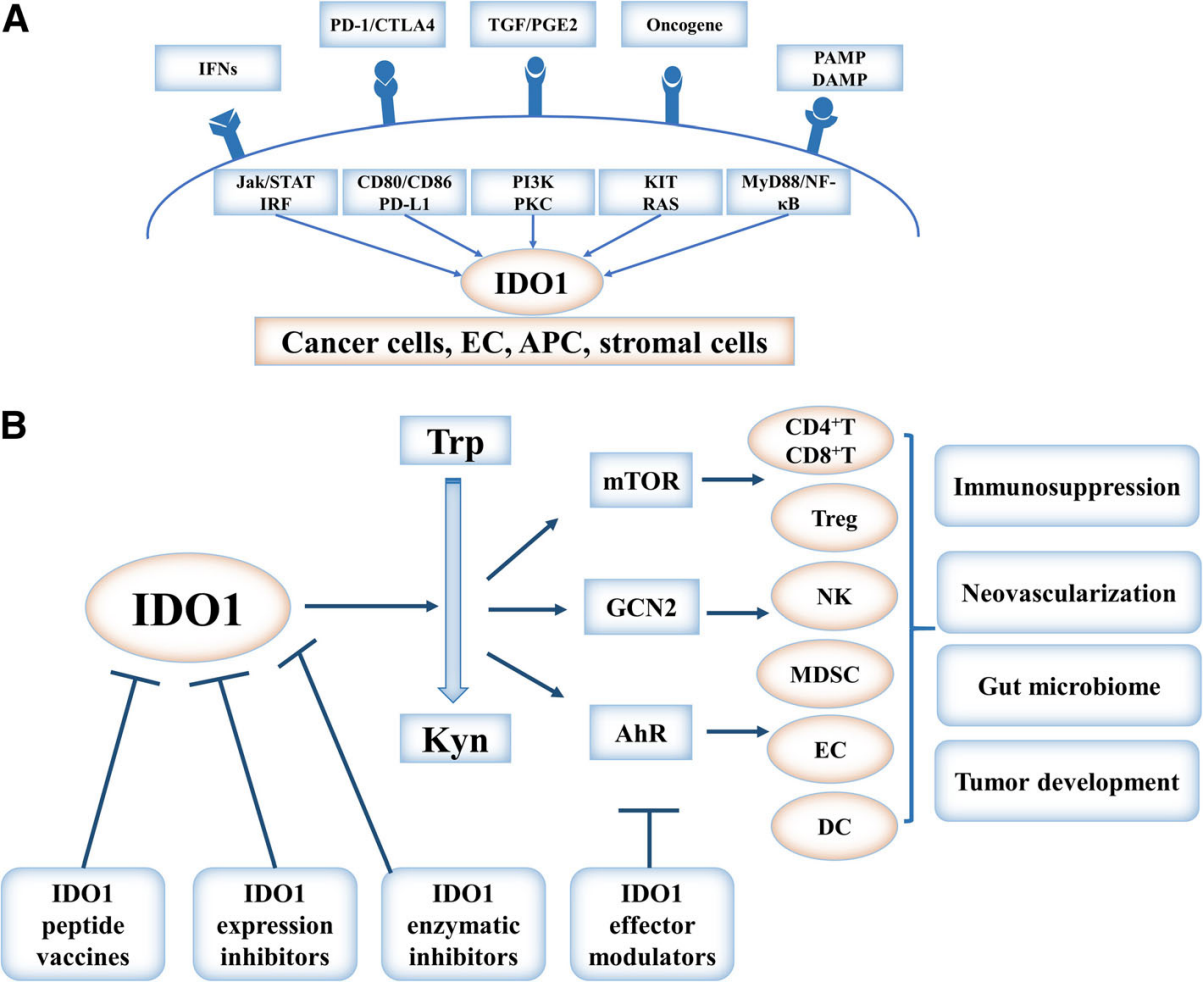
Regulation, function, and targeting of IDO1 in cancer. **a** The upstream regulators of IDO1. IDO1 is expressed in cancer cells, endothelial cells, antigen-presenting cells, and stromal cells. IDO1 expression is regulated by IFNs, PD-1, oncogene activation (KIT or RAS), PAMPs, and DAMPs through relevant signaling pathways like IFN-γ/JAK/STAT, PI3K/PKC, and NF-κB. **b** The downstream effectors of IDO1 and IDO1 targeting. Three effector pathways (mTOR, GCN2, and AhR) mediate the effects of IDO1 activities in various types of cells in regard to immunosuppression, neovascularization, interactions with the gut microbiome, and the tumor microenvironment. Four strategies have been developed to target the IDO1 pathway in preclinical and clinical studies. IDO1, indoleamine 2, 3-dioxygenase 1; Trp, tryptophan; Kyn, kynurenine; IFN, interferon; PD-1, programmed death receptor 1; PAMP, pathogen-associated molecular pattern; DAMP, damage-associated molecular pattern; GCN2, general control over nonderepressible 2; mTOR, mammalian target of rapamycin; AhR, aryl hydrocarbon receptor; NK, natural killer cell; Treg, regulatory T cell; MDSC, myeloid-derived suppressor cell; EC, endothelial cell; DC, dendritic cell

IDO1 is not expressed in most tissues in adult humans under physiological conditions but is constitutively expressed in many types of cancer cells, stromal cells, and immune cells in the tumor microenvironment (Fig. 3a). IDO1 is activated by diverse inflammatory molecules, such as IFN-γ, tumor necrosis factor α (TNF-α), transforming growth factor β (TGF-β), pathogen-associated molecular patterns (PAMPs), damage-associated molecular patterns (DAMPs), and prostaglandin E2 (PGE2), through canonical and non-canonical NF-κB and JAK–STAT pathways [21, 23, 25–27]. Constitutive IDO1 expression in human cancers is driven by cyclooxygenase 2 (COX-2) and PGE2 via the PKC and PI3K pathways [28]. Moreover, autocrine TGF-β sustains the activation of IDO1 in a tolerogenic subpopulation of CD8^+^ dendritic cells (DCs), while exogenous TGF-β converts immunogenic CD8^−^ DCs into tolerogenic cells in conjunction with induction of IDO1 [29]. IDO1 is under genetic control of the cancer-suppression gene *bridging integrator 1* (Bin1), and *Bin1* knockout results in tumor growth and immune suppression in mice by upregulating STAT1- and NF-κB-dependent expression of IDO1 [20]. Ras and Kit also upregulate IDO1 expression in cancer cells [22, 27]. Importantly, several immune checkpoints (e.g., PD-1 and CTLA4) on the T cell surface modulate IDO1 expression in antigen-presenting cells. Recently, Wang et al. [30] demonstrated that the IL-6-inducible proto-oncogene protein intestine-specific homeobox (ISX) gene induces IDO1 and TDO expression, which increases Kyn and AhR and thereby promotes the tumorigenic potential and immunosuppression of hepatocellular carcinoma cells expressing CD86 and PD-L1. Others report that hypoxia enhances IDO1 production in monocyte-derived dendritic cells, yet the underlying mechanisms remain elusive [31].

### Downstream effectors

IDO1 transduces signaling through three major effectors: GCN2, mTOR, and AhR. The depletion of Trp by IDO1 leads to the accumulation of uncharged Trp–tRNA, which binds and activates GCN2, a stress-response kinase. Activated GCN2 phosphorylates and inhibits eukaryotic initiation factor 2α kinase, resulting in attenuation of RNA transcription and protein translation. Specific to T effector cells, GCN2 activation mediated by Trp deprivation leads to cell cycle arrest and/or apoptosis [25, 32]. Another signaling molecule inhibited by Trp deprivation is the mammalian target of rapamycin (mTOR) [23]. IDO1-mediated suppression through mTORC1 triggers autophagy, leading to anergy in T cells in the tumor microenvironment [23, 33]. Most relevant publications report that mTOR inhibition suppresses effector T cell function and promotes Treg cell function although others dispute these findings [34], and the precise role of IDO1 in mTOR signaling and immune regulation continues to be debated. Both GCN2 kinase activation and mTOR inhibition are immunosuppressive. During CD4^+^ T-cell differentiation, GCN2 kinase activation suppresses only Th2 differentiation, whereas mTOR inhibition induces Treg cell differentiation and suppresses differentiation of the Th1, Th2, and Th17 lineages [35]. These findings suggest that Trp deprivation-mediated GCN2 kinase activation and mTOR inhibition have differential impacts on the function of distinct CD4^+^ T cell populations. AhR is a cytosolic ligand-activated transcription factor involved in embryogenesis, adaptive immunity, mucosal barrier function, and malignancy. Its most potent ligand, 2,3,7,8-tetrachlorodibenzo-p-dioxin (TCDD), activates AhR signaling to upregulate IDO1 expression [36]. A breakthrough was reported in 2011 in which Kyn was found to be an endogenous ligand of AhR [37]. The binding of Kyn to AhR promotes naive CD4^+^ T cell differentiation into Treg cells, which contributes to an immunosuppressive tumor microenvironment [38].

## Role of the IDO1 pathway in cancers

IDO1 is expressed by various cancer and cancer-associated cells in the tumor microenvironment, including DCs, endothelial cells, tumor-associated macrophages, tumor-associated fibroblasts, mesenchymal stromal cells (MSCs), and MDSCs [2, 39]. Most cancer types have high IDO1 expression (Fig. 2), which is correlated with poor survival and prognosis [40–42]. Besides its role in immunosuppression, IDO1 also contributes to cancer development by promoting inflammatory neovascularization [27], interacting with checkpoint inhibitors, and modulating gut microbiota [43].

### IDO1 ablates T effector cells and promotes the induction of Treg cells and MDSCs in the tumor microenvironment

Increased IDO1 and accumulating Trp metabolites prevent the activation of CD8^+^ and CD4^+^ effector T cells, inhibit NK cell function, stimulate the activation of Treg cells, and promote the expansion and activation of DCs and MDSCs [25, 44–46]. Treg cells are a major suppressive cell type in the tumor microenvironment, as their recruitment is induced by high IDO1 levels and correlates with poor prognosis in several tumor types [47]. Furthermore, IDO1 presence leads to activation of the phosphatase and tensin homolog (PTEN) pathway in Treg cells to maintain their immunosuppressive phenotype in vitro [48] .

IDO1 is also essential for the recruitment and sustenance of MDSCs through a Treg-dependent mechanism [49]. MDSCs can suppress antitumor immune responses through STAT3-dependent IDO1 expression in breast cancer [50]. Increased STAT3 activation in MDSCs was found to be correlated with activation of the non-canonical NF-κB pathway, in which RelB–p52 dimers directly bind to the IDO1 promoter, leading to IDO1 expression in MDSCs [51]. In a Kras mutant lung cancer model, *Ido1*-deficient mice had impaired MDSC function [27].

Cancer-associated fibroblasts recruit and educate DCs to become IDO1-producing regulatory DCs through IL-6-mediated STAT3 activation [52]. Mesenchymal stem/stromal cells in the tumor microenvironment promote tumor growth through IDO1-mediated immune suppression [53]. Expression of IDO1 in tumor endothelial cells is negatively associated with long-term survival in patients with renal cell carcinoma [54]. In addition, the IDO1 expression level in tumor endothelial cells is associated with the tumor’s response to checkpoint inhibitor treatment in metastatic renal cell carcinoma [55].

### IDO1 and immune checkpoint inhibitors

Antibodies targeting cytotoxic T cell antigen 4 (CTLA-4), programmed cell death 1 (PD-1), and programmed cell death ligand 1 (PD-L1) have been approved by the Food and Drug Administration (FDA) for multiple cancers [56, 57]. Recent research further revealed that IDO1 is closely linked to both CTLA-4 and PD-1–PD-L1 via complex pathways. Treg cell-expressed CTLA-4 induced IDO1 expression in DCs [46], and PD-1/PTEN signaling in IDO1-activated Treg cells was required to maintain immune suppression of the Tregs [58]. IDO1 expression in DCs is induced by the interaction of PD-1 with PD-L1 on the surface of mast cells and/or with PD-L2 on the surface of DCs [59]. Moreover, IDO1 participates in the intracellular signaling events responsible for long-term tolerance by DCs [29]. Based on its upstream effects on DCs, this IDO1 activity is non-redundant with that of the more distal T cell checkpoints, and combined therapy with IDO1 inhibitors and CTLA-4 or PD-1 inhibitors should confer additional benefit.

IDO1 has also been reported to participate in a resistance mechanism to checkpoint inhibitors, and the combination of CTLA-4 blockade and an IDO1 inhibitor resulted in more effective antitumor immunity in a melanoma mouse model [13]. In a phase 2 trial, excessive infiltration of IDO1^+^ macrophages and a significant increase in the kynurenine-to-Trp ratio were observed in the majority of patients who had poor response to metronomic cyclophosphamide and pembrolizumab combination treatment [60]. In addition, Liu and colleagues identified a Kyn–AhR pathway-dependent mechanism that promoted tumor-repopulating cell immune escape by increasing PD-1 binding to CD8^+^ T cells. Local IFN-γ produced by tumor-infiltrating CD8^+^ T cells stimulates tumor-repopulating cell release at high levels of Kyn, which then activates AhR on the T cell surface to promote PD-1 upregulation. Thus, targeting the Kyn–AhR pathway (such as by inhibiting IDO1 in tumor-repopulating cell or AhR in CD8^+^ T cells) may enhance the efficacy of adoptive T cell therapy. Taken together, these results suggest that combination strategies targeting multiple immune checkpoints might be the preferred weapon of the future.

### Tumor neovascularization

Neovascularization, characterized by excessive and disorganized growth of blood vessels, is critical for tumor development, progression, and metastasis. In mice, *Ido1* deficiency inhibited lung tumor growth and improved animal survival with reduced density of the underlying pulmonary blood vessels [27]. Recent work also showed a new role for IDO1 in supporting pathologic neovascularization [11]. In 4T1 breast cancer pulmonary metastases and oxygen-induced retinopathy mouse models, IDO1 ablation was sufficient to reduce pathological neovascularization in both lung metastases and reinopathy. IL-6 is regarded as a pro-angiogenic cytokine potentiated by IDO1, and IL-6 deletion results in IFN-γ-dependent reduction in neovascularization and increases resistance to metastasis [11]. Administration of the IDO1 inhibitor epacodostat (INCB024360) in mice with tumors or vascularized metastases significantly reduced neovascularization [11]. High IDO1 expression positively correlates with microvessel density and poor prognosis in breast cancer patients [61]. In summary, IDO1-mediated effects on neovascular development and established neovascular networks broaden the potential effectiveness of IDO1 inhibitors in clinical trials and in practice.

### IDO1 and the gut microbiome

The gut microbiome has been found to regulate cancer initiation, progression, and response to therapies [62]. Preclinical mouse models suggest that resistance in melanoma patients to anti-PD-1 immunotherapy can be attributed to abnormal gut microbiome composition [63]. Bacteria, including *Bifidobacterium longum*, *Collinsella aerofaciens*, *and Enterococcus faecium*, are more abundant in treatment responders, whereas the efficacy of immune checkpoint blockade therapies is diminished with administration of antibiotics [64–66]. As an essential nutrient in mammals, Trp and its IDO1-catalyzed endogenous metabolites play a key role in the gut microbiota and immune homeostasis. Recent discoveries have underscored the modulation of host immune systems by changes in the microbiota that affect Trp metabolism [43]. Activation of toll-like receptors by microbial components has been identified as a key factor in initiating Kyn metabolism and gut microbial homeostasis, as reduced toll-like receptor stimulation in germ-free mice resulted in decreased Trp metabolism [53, 67].

## IDO1 inhibitors in preclinical development and clinical trials

Based on the important role of IDO1 in cancer immune tolerance and development, targeting IDO1 is becoming an attractive approach in cancer therapeutic development (Fig. 3b). Unlike cell-surface checkpoint receptor molecules that can be effectively targeted by antibody-based therapeutics, IDO1 and its downstream effector molecules are intracellular targets that are still best addressed by small molecule drugs. An increasing number of IDO1 inhibitors are being tested in preclinical development or clinical trials [15, 16] (Table 1).

**Table 1:** IDO1 inhibitors in clinical trials

Drug	Strategies	Tumor type	Phase	Clinical efficacy	Safety (% patients)	Trial number	Status
Indoximod	Single agent	Metastatic or refractory solid tumors	I	NR	NR	NCT00739609	Terminated
		Metastatic or refractory solid tumors	I	ORR 10% (5/48)	Fatigue (56.3%), anemia (37.5%), anorexia (37.5%) dyspnea (35.4%) cough (33.3%) nausea (29.2%)	NCT00567931	Completed
	Docetaxel	Metastatic solid tumors	I	4/22PRs, 9/22 SD, and 9/22 PD	Fatigue (58.6%) anemia (51.7%) hyperglycemia (48.3%), infection (44.8%), nausea (41.4%)	NCT01191216	Completed
	Temozolomide/bevacizumab	Primary malignant brain tumors	I/II	NR	NR	NCT02052648	Recruiting
	Temozolomide	Progressive primary malignant brain tumors	I			NCT02502708	Recruiting
	Docetaxel/paclitaxel	Metastatic breast cancer	II			NCT01792050	Unknown
	Nab-Paclitaxel/gemcitabine	Metastatic pancreatic cancer	I/II	ORR 11/30 (37%)	1/30 (colitis)	NCT02077881	Recruiting
	Idarubicin/cytarabine	Acute myeloid leukemia	I/II			NCT02835729	Recruiting
	Adenovirus-p53 transduced dendritic cell (DC) vaccine	Metastatic breast cancer	I/II	Chemosensitization effect, median PFS 13.3 weeks and median OS 20.71 weeks. 9/22 patients benefitted from chemotherapy after vaccination.	Most common grade 1–2 (fatigue, anemia, transient lymphopenia, nausea, anorexia)	NCT01042535	Completed
	Sipuleucel-T	Refractory metastatic prostate cancer	II	NR	NR	NCT01560923	Active, not recruiting
	Tergenpumatucel-L/docetaxel	Advanced previously treated non-small cell lung cancer	I/II			NCT02460367	Unknown
	Ipilimumab/nivolumab/pembrolizumab	Metastatic melanoma	II/ III			NCT03301636	Recruiting
Epacadostat	Single agent	Advanced malignancy	I/II	NR	Fatigue, nausea, decreased appetite, vomiting, constipation, abdominal pain, diarrhea, dyspnea, back pain, cough	NCT01195311	Completed
		Solid tumor	I			NCT03471286	Not yet recruiting
		Myelodysplastic syndromes	II			NCT01822691	Completed
	Fludarabine/cyclophosphamide/NK cells/IL-2	Recurrent ovarian, fallopian tube, and primary peritoneal cancer	I			NCT02118285	Completed
	Tamoxifen	Ovarian cancer genitourinary tumors	II	Median PFS, 3.75 months	Fatigue (36.4%) rash (18.2%) pruritus (9.1%)	NCT01685255	Terminated
	Azacitidine + pembrolizumab	Advanced solid tumors	I/II	NR	NR	NCT02959437	Recruiting
	MELITAC 12.1 peptide vaccine	Stage III-IV melanoma	II	NR	NR	NCT01961115	Active, not recruiting
	DPX-survivac/cyclophosphamide	Ovarian cancer	I	NR	NR	NCT02785250	Recruiting
	ALVAC(2)-NY-ESO-1 (M)/TRICOM vaccine	Ovarian, fallopian tube, or primary peritoneal cancer	I/II	NR	NR	NCT01982487	Withdrawn
	DEC-205/NY-ESO-1 fusion protein CDX-1401/poly ICLC	Ovarian, fallopian tube, or primary peritoneal cancer	I/II	NR	NR	NCT02166905	Recruiting
	CRS-207/pembrolizumab	Metastatic pancreas cancer, platinum-resistant ovarian, fallopian, or peritoneal cancer	II,I/II	NR	NR	NCT03006302	Recruiting active, not recruiting
						NCT02575807	
	Atezolizumab	Non-small cell lung cancer and urothelial carcinoma	I	NR	NR	NCT02298153	Terminated
	Durvalumab	Advanced solid tumor	I/II	NR	NR	NCT02318277	Recruiting
	Ipilimumab	Melanoma	I/II			NCT01604889	Terminated
	Nivolumab/ipilimumab/lirilumab	Solid tumors	I/II			NCT03347123	Recruiting
	Nivolumab/chemotherapy	Advanced cancers	I/II	ORR 75%(melanoma) 11% (ovarian) 4% (colorectal)	Rash (10% and 12% in epacadostat 100 and 300 mg subgroups)	NCT02327078	Recruiting
	Pembrolizumab/chemotherapy	Advanced solid tumors	I, I/II	NR	NR	NCT02862457	Recruiting
						NCT03085914	Recruiting
	pembrolizumab	Solid tumors, thymic carcinoma, sarcoma, junction or gastric cancer, lung cancer, urothelial cancer, metastatic melanoma, and others.	I, I/II, III	Melanoma (ORR 57% and DCR 86%), Renal cell carcinoma (ORR 40% and DCR 80%)	Fatigue, diarrhea, rash, arthralgia, and nausea	NCT02178722	Recruiting
						NCT02364076	Recruiting
						NCT03414229	Recruiting
						NCT03196232	Recruiting
						NCT03322540	Recruiting
						NCT03361865	Recruiting
	Pembrolizumab	Melanoma	III			NCT02752074	Halted
Navoximod	Single agent	Advanced solid tumors	I	NR	NR	NCT02048709	Completed
	Atezolizumab	Solid tumors	I			NCT02471846	Active, not recruiting
PF-06840003	Single agent	Malignant gliomas	I			NCT02764151	Active, not recruiting
BMS-986205	Nivolumab	Melanoma advanced cancers	III, I/II	NR	3/42 patients with grade 3 autoimmune hepatitis	NCT03329846	Recruiting
						NCT03192943	Recruiting
	Nivolumab/ipilimumab	Advanced cancer melanoma non-small cell lung cancer	I/II	ORR 32%(bladder cancer) 14%(cervical cancer) PD-L1 > 1%: 46% (bladder cancer)and 25% (cervical cancer)	Fatigue (18.2% nausea (18.2%) decreased appetite (13.6%) vomiting (6.8%)	NCT02658890	Recruiting
	Nivolumab/ipilimumab/relatlimab	Advanced gastric cancer, advanced renal cell carcinoma, advanced cancer	II, I/II	NR	NR	NCT02935634	Recruiting
						NCT02996110	Recruiting
						NCT03459222	Not yet recruiting
						NCT03335540	Recruiting
	Nivolumab/cetuximab/chemotherapy	Head and neck cancer	III	NR	NR	NCT03386838	Halted
	Nivolumab/chemotherapy	Lung cancer	III			NCT03417037	Halted

### The effector inhibitor indoximod

Indoximod (1-methyl-D-tryptophan, 1MT, NLG-8189) is the most-studied IDO1 inhibitor and has been granted orphan-drug designation by the US FDA for the indication of stage IIb to stage IV melanoma. Recent results suggest that indoximod is not only a valid inhibitor of IDO1 enzymatic activity but also acts as a high-potency Trp mimetic in reversing mTORC1 inhibition [23]. mTORC1 is a central integrator of cell growth signals that monitors levels of essential amino acids that are needed to activate cell growth [68]. Clinical results indicated that indoximod exerted little antitumor efficacy as a single agent, but efficacy was markedly enhanced when it was combined with other therapies, such as PD-1 checkpoint inhibitors (in advanced melanoma), cancer vaccines (in metastatic prostate cancer), and chemotherapy (in pancreatic cancer and acute myeloid leukemia). A phase 1 dose-escalation trial aiming to evaluate the safety, dosing, pharmacokinetics, and immunologic effects of indoximod found indoximod to be safe at doses up to 2000 mg orally twice daily [69]. Another phase 1 dose-escalation trial designed to study co-administration of docetaxel and indoximod reported that this combination is well tolerated, with no increase in expected toxicities in patients with metastatic solid tumors [70]. Administering indoximod with the PD-1 antibody pembrolizumab (Keytruda) led to a 61% overall response rate, including 10 complete responses (20%) and 21 partial responses (41%), in patients with advanced melanoma. The median progression-free survival under combinatorial treatment was 12.9 months, with a 1-year rate of 56%, suggesting a synergistic antitumor therapeutic effect [71].

### IDO1 enzymatic inhibitors

#### Epacadostat (INCB024360)

Epacadostat is a Trp competitive inhibitor that has been widely investigated in clinical trials. With an IC50 of 72 nM, it has a > 1000-fold selectivity for the IDO1 enzyme relative to IDO2 or TDO [72]. In vitro studies found that epacadostat promoted T cell and NK cell proliferation, increased the number of CD86^high^ DCs, and reduced Treg cells [73], and its administration to tumor-bearing syngeneic mice inhibited plasma and tumor Kyn levels by approximately 90% and reduced tumor growth in immunocompetent but not immunocompromised mice.

In a phase 1 clinical study, epacadostat was well tolerated at doses of 100 mg twice daily. No objective responses were detected although stable disease was observed in 7 of 52 patients over 16 weeks of observation [74]. A randomized phase 2 study compared epacadostat with tamoxifen treatment in 42 patients with biochemically recurrent epithelial ovarian cancer, primary peritoneal carcinoma, or fallopian tube cancer, found that epacadostat was well tolerated but its efficacy was no better than tamoxifen [75]. Based on encouraging results from its combination with immune checkpoint inhibitors, epacadostat was propelled into three clinical trials [76]. In melanoma, anti-PD-1 antibody combinations (either pembrolizumab or nivolumab) showed rates of overall response and disease control similar to those produced by the approved combination of anti-PD-1 and anti-CTLA-4 antibodies (ipilimumab) without the significant side effects of the latter. In head and neck cancer, an interim report on 38 patients who were previously treated suggested that epacadostat increased rates of overall response and disease control without any notable increase in side effects when administered with an anti-PD-1 antibody. Epacadostat is currently being tested in clinical trials in 14 tumor types (including the above cancers) with co-administration of anti-PD-1 antibodies (nivolumab or pembrolizumab) or anti-PD-L1 antibodies (atezolizumab and durvalumab).

#### Navoximod (NLG-919, GDC-0919)

Navoximod was initially developed as an orally bioavailable IDO1 and TDO inhibitor with a superior pharmacokinetic and toxicity profile based on 4-phenylimidazole, a compound that binds the heme iron at the IDO1-active site [77]. In preclinical studies, navoximod inhibited IDO1-induced T cell suppression and restored robust T cell responses (EC50 = 80 nM). Kyn levels in plasma were reduced by approximately 50% in mice treated with navoximod [78]. In a syngeneic murine B16-F10 melanoma model, navoximod potentiated the antitumor efficacy of paclitaxel without increasing side effects [79]. A combination of navoximod, anti-PD1/PD-L1/PD-L2 antibodies, and indoximod with chemotherapy and a glycoprotein 100 (gp100) peptide vaccine achieved a significant synergistic antitumor effect [80]. In a phase 1b clinical trial, the combination of navoximod and atezolizumab was well tolerated [81]; preliminary efficacy data from 45 patients with over 1 on-treatment tumor assessments included 4 patients (9%) with partial response and 11 (24%) patients with stable disease.

#### BMS-986205

BMS-986205 is an irreversible IDO1 inhibitor. In preclinical studies, BMS-986205 was found to specifically target and bind to IDO1 but not IDO2 or TDO [15]. By inhibiting IDO1 and decreasing Kyn levels in tumor cells, BMS-986205 reversed immunosuppression in cancer patients. In a phase 1/2a study, BMS-986205 was administered alone or in combination with nivolumab in multiple advanced malignancies. It was well tolerated, with no grade 3 events in BMS-986205 monotherapy and no grade 4 or 5 events in the combination group. In a dose escalation and expansion study (NCT02658890), an encouraging response was observed for BMS-986205 plus nivolumab. The maximum tolerated dose of BMS-986205 in combination with nivolumab was 200 mg, and the recommended dose for further study was 100 mg. Objective response rates in the bladder and cervical cancer cohorts were 32% and 14%, respectively, which improved to 46% and 25%, respectively, in tumors where over 1% of tumor cells expressed PD-L1. An increase in tumor-infiltrating CD8^+^ T cells and a decrease in Kyn were also observed [82]. Based on this potent effect and encouraging results overall, about 10 ongoing trials are investigating the effect of BMS-986205 combined with nivolumab as compared with nivolumab alone in patients with advanced melanoma, non-small cell lung cancer, head and neck cancer, advanced gastric cancer, and other types of cancer.

#### PF-06840003 and other inhibitors

PF-06840003 is an orally bioavailable, highly selective IDO1 inhibitor that can cross the blood–brain barrier. In vitro, it reverses IDO1-induced T-cell anergy. In preclinical syngeneic mouse tumor models, PF-06840003 reduced Kyn levels in mice by > 80% and enhanced the antitumor efficacy of anti-PD-1 or anti-PD-L1 antibodies. This compound has favorable human pharmacokinetic characteristics, with a prolonged half-life that enables single-dose daily administration. More importantly, its central nerve system (CNS) penetration properties allow its application in brain metastases [83]. An ongoing multi-center clinical trial aims to assess the safety, pharmacokinetic, and pharmacodynamic activity of PF-06840003 in malignant glioma and to validate its CNS penetration and effectiveness in combination with other drugs. Recently, BGB-5777, a potent CNS-penetrating IDO1 inhibitor, when combined with nivolumab and radiation therapy, achieved a durable survival benefit in patients with advanced glioblastoma [17]. A few other IDO1 inhibitors are in preclinical development, including Trp analogs, imidazoles, phenyl benzenesulfonylhydrazides, N-hydroxyamidines, and tryptanthrin derivatives [84]. These compounds offer novel scaffolds for central nerve system (CNS) optimization, providing abundant possibilities for developing highly specific IDO1 inhibitors.

### IDO1 peptide vaccines

Previous studies have found that IDO1 peptides elicit specific CD8^+^ T cells that recognize and kill IDO1-expressing tumor cells and DCs and simultaneously enhance other T-cell responses [85]. In a phase 1 trial to evaluate the efficacy and safety of IDO1 vaccines, 15 patients with advanced NSCLC were injected with an IDO1-derived human leukocyte antigen A2-restricted epitope. The median overall survival was 25.9 months with no grade 3 or 4 toxicities. Furthermore, all treated patients had a significantly reduced Treg cell population after the sixth dose of vaccine [86]. Based on these promising results and distinct mechanisms of action, an additional phase 1 trial combining IDO1 vaccines with CTLA-4 inhibitor ipilimumab (Yervoy) for stage III or IV melanoma patients and a phase 1/2 clinical trial that tests a combination treatment with a PD-1 monoclonal antibody (nivolumab) and a PD-L1–IDO1 peptide vaccine, have been initiated. Other potential strategies, such as combining IDO1 inhibitors with IDO1 vaccines may also produce synergistic anti-tumor effects [87].

### IDO1 expression inhibitors

Gene expression is often repressed by microRNAs. Our group analyzed IDO1 downregulation by microRNA-153 (miR-153) in colon cancer cells and the association of IDO1 and miR-153 expression with colorectal patient survival [18]. We found that IDO1 is highly expressed in colorectal tumors and is inversely associated with patient survival. miR-153 directly inhibits IDO1 expression by targeting its 3′ untranslated region in colon cancer cells, yet miR-153 overexpression does not affect colon cancer cell survival, apoptosis, or colony formation. When colon cancer cells are targeted by chimeric antigen receptor (CAR) T cells, miR-153 overexpression within tumor cells significantly enhances T cell killing in vitro and suppresses xenograft tumor growth in mice. These findings indicate that miR-153 is a tumor-suppressive miRNA that enhances CAR T cell immunotherapy and supports the combinatorial use of IDO1 inhibitors and CAR T cells in treating solid tumors [18].

## Conclusions

IDO1 has diverse biological roles in immune suppression and tumor progression, rendering it an attractive target in cancer therapeutic development. IDO1 inhibitors may serve as an “immunometabolic” adjuvant to enhance systemic immune responses and turn immunologically “cold” tumors “hot.” Targeting IDO1 represents a therapeutic opportunity in cancer immunotherapy beyond checkpoint blockade [88–90] or adoptive transfer of CAR T cells [91–95]. Although IDO1 inhibitor monotherapies have shown disappointing efficacy, combinations of IDO1 inhibitors with conventional treatments show satisfactory efficacy in several trials. We note that IDO1 inhibitors suffered major setbacks in recent clinical trials. For the pivotal phase 3 ECHO-301/KEYNOTE-252 clinical trial (NCT02752074) that evaluated epacadostat in combination with pembrolizumab in patients with unresectable or metastatic melanoma, the study did not meet the primary endpoint of improving progression-free survival in the overall population compared with pembrolizumab monotherapy [96]. Two phase 3 trials (NCT03386838 and NCT03417037) evaluating the IDO1 inhibitor BMS-986205 in combination with nivolumab were also halted after re-evaluation. These negative results underscore that much about IDO1’s role in immune suppression remains unresolved. Further studies on the basic biology of IDO1/2 and TDO are essential to guide clinicians in identifying drug combinations that are more effective and selecting patients who are most likely to benefit from them. IDO1’s role in neovascular development suggests that when clinical results of IDO1 inhibitors are assessed, the impact on tumor vasculature should be examined. Targeting IDO2 and TDO in addition to IDO1 may open new windows for cancer immunotherapy.

## Abbreviations

AhR: Aryl hydrocarbon receptor
CAF: Cancer-associated fibroblast
CAR: Chimeric antigen receptor
CNS: Central nerve system
CTLA-4: Cytotoxic T cell antigen 4
DAMP: Damage-associated molecular pattern
DC: Dendritic cell
DCR: Disease control rate
GCN2: General control over nonderepressible 2
IDO: Indoleamine 2, 3-dioxygenase
IFN-γ: Interferon-γ
JAK: Janus kinase
Kyn: Kynurenine
MDSC: Myeloid-derived suppressor cell
MSC: Mesenchymal stromal cell
mTOR: Mammalian target of rapamycin
NF-κB: Nuclear factor kappa-light-chain-enhancer of activated B cells
NR: Not reported
ORR: Objective response rate
OS: Overall survival
PAMP: Pathogen-associated molecular pattern
PD: Progressive disease
PD-1: Programmed death receptor 1
PD-L1: Programmed death receptor ligand 1
PFS: Progression-free survival
PGE2: Prostaglandin E2
PKC: Protein kinase C
PR: Partial response
SD: Stable disease
STAT: Signal transducer and activator of transcription
TAM: Tumor-associated macrophage
TDO: Tryptophan 2,3-dioxygenase
TGF-β: Transforming growth factor β
TNF-α: Tumor necrosis factor α
Treg: Regulatory T cell
Trp: Tryptophan
